# Mechanisms of scaling up: combining a realist perspective and systems analysis to understand successfully scaled interventions

**DOI:** 10.1186/s12966-021-01103-0

**Published:** 2021-03-22

**Authors:** Harriet Koorts, Samuel Cassar, Jo Salmon, Mark Lawrence, Paul Salmon, Henry Dorling

**Affiliations:** 1 Deakin University, Institute for Physical Activity and Nutrition (IPAN), School of Exercise and Nutrition Sciences, Geelong, VIC Australia; 2 Centre for Human Factors and Sociotechnical Systems, Faculty of Arts, Business and Law, University of the Sunshine Coast, Queensland, Australia; 3 Solent University, School of Sport, Health and Social Science, Southampton, Hampshire UK

**Keywords:** Scale up, Systems, Physical activity, Nutrition, Intervention, Realist evaluation, Implementation

## Abstract

**Background:**

Sustainable shifts in population behaviours require system-level implementation and embeddedness of large-scale health interventions. This paper aims to understand how different contexts of scaling up interventions affect mechanisms to produce intended and unintended scale up outcomes.

**Methods:**

A mixed method study combining a realist perspective and systems analysis (causal loop diagrams) of scaled-up physical activity and/or nutrition interventions implemented at a state/national level in Australia (2010–18). The study involved four distinct phases: ***Phase 1*** expert consultation, database and grey literature searches to identify scaled-up interventions; ***Phase 2*** generating initial Context-Mechanism-Outcome configurations (CMOs) from the WHO ExpandNet framework for scaling up; ***Phase 3*** testing and refining CMOs via online surveys and realist interviews with academics, government and non-government organisations (NGOs) involved in scale up of selected interventions (*Phase 1*); and ***Phase 4*** generating cross-case mid-range theories represented in systems models of scaling up; validated by member checking. Descriptive statistics were reported for online survey data and realist analysis for interview data.

**Results:**

Seven interventions were analysed, targeting nutrition (*n* = 1), physical activity (n = 1), or a combination (*n* = 5). Twenty-six participants completed surveys; 19 completed interviews. Sixty-three CMO pathways underpinned successful scale up, reflecting 36 scale up contexts, 8 key outcomes; linked via 53 commonly occurring mechanisms. All five WHO framework domains were represented in the systems models. Most CMO pathways included ‘intervention attributes’ and led to outcomes ‘community sustainability/embeddedness’ and ‘stakeholder buy-in/perceived value’. Irrespective of interventions being scaled in similar contexts (e.g., having political favourability); mechanisms still led to both intended and unintended scale up outcomes (e.g., increased or reduced sustainability).

**Conclusion:**

This paper provides the first evidence for mechanisms underpinning outcomes required for successful scale up of state or nationally delivered interventions. Our findings challenge current prerequisites for effective scaling suggesting other conditions may be necessary. Future scale up approaches that plan for complexity and encourage iterative adaptation throughout, may enhance scale up outcomes. Current linear, context-to-outcome depictions of scale up oversimplify what is a clearly a complex interaction between perceptions, worldviews and goals of those involved. Mechanisms identified in this study could potentially be leveraged during future scale up efforts, to positively influence intervention scalability and sustainability.

**Supplementary Information:**

The online version contains supplementary material available at 10.1186/s12966-021-01103-0.

## Background

Characterised by the World Health Organization (WHO) as the ‘epidemic of the 21st century’, obesity remains one of the most serious global public health challenges to date [1]. Obesity and being overweight are leading risk factors for cardiovascular disease, type 2 diabetes, and numerous cancers [1]. Having a poor diet and low levels of physical activity are leading contributors to the global burden of disease [2]. Australia is one of the most overweight developed nations, ranked fifth highest among Organisation for Economic Co-operation and Development countries [3], with an estimated 67% of adults and one in four children classified overweight or obese [4]. Only 43% of adults, 29% of children (5–11 years) and 8% of adolescents (12–17 years) achieve government recommended levels of physical activity for health (2011–12) [5]. On average, men and women of all ages and the majority of adolescents fail to consume the recommended number of serves for any of the nutritious five food group food categories, whereas discretionary food consumption contributes 33 to 36% and 41% to daily energy intake for these population groups, respectively [6]. Sustainable shifts in population behaviours require system-level implementation and embeddedness of large-scale effective health interventions or programs, and yet a minority of efficacious interventions are translated into practice and delivered at a scale sufficient to achieve population-wide effects [7]. The WHO has long emphasised the importance of accelerating the impact of interventions at scale [8, 9]. Despite some evidence for the potential benefits of large scale physical activity, nutrition and obesity prevention programs in Australia and globally [10], there has been little research of the kind that is needed to inform delivery of interventions at scale to achieve population-level impacts in Australia [11].

There is evidence that scale up strategies (e.g., dissemination via strategic advocates/champions) may be more successful for some interventions in some contexts, and approaches may need to be systematic, involve a range of stakeholders, and be adaptive to the local setting [12]. However, studies often fail to report important details, including the *goals* of scale up (e.g., beyond health impact), *how* scale up was achieved (e.g., drivers and mechanisms influencing outcomes), and *who* did what (e.g., the activities and impact of different actors). To date, knowledge of scaling efficacious physical activity and nutrition interventions has focussed on the *factors associated* with effective implementation and scale up [13, 14], and the *process* of moving from efficacy to at-scale implementation (e.g., [15]). However, there is a lack of evidence for ways of overcoming methodological challenges when assessing the potential scalability of interventions in terms of optimal timing, consensus on the purpose of scaling, and trade-offs between intervention design for controlled trials and that which is appropriate for scale up [16].

What has yet to be investigated are the potential *mechanisms* underpinning successful scale up, and their related ‘leverage points’ and how these mechanisms differ by context to produce observed outcomes. There are known leverage points that are relevant for changing food and physical activity environments; ‘leverage points’ are places within a complex system whereby a small shift in one aspect can lead to significant changes in another [17]. For example, the availability of recreation facilities is a proximal leverage point and laws governing food systems/industry is a distal leverage point [18]; however, these are complex public health issues driven by multiple determinants [19–21] that are embedded in complex social and political systems. Understanding and identifying mechanisms linked to specific outcomes of physical activity, nutrition and obesity prevention programs when scaled, is essential to help target potential leverage points for improving the effectiveness of implementation efforts and potential health impacts of interventions. Disaggregating why such complex interventions work, how, for whom and in what context, and explaining why an intervention may fail to achieve anticipated benefits or fail to be successfully scaled is fundamental to a realist perspective [22].

A realist perspective assumes that an intervention may lead to different *outcomes* in different *contexts* due to different *mechanisms*. This leads to ‘Context–Mechanism–Outcome’ (CMO) configurations, which are essentially a hypothesis about how an intervention works in a particular context [23]. CMO configurations are the basic causal explanatory framework for realist evaluation, and unlike ‘factors’ which are related to change but need not be causal, ‘mechanisms’ produce change. The use of systems approaches, such as causal loop diagrams [24], to pictorially demonstrate these CMOs provides a useful visual tool to help illustrate the assumptions, and links that influence behaviour [25]. Although systems methodologies have been recommended when trying to identify and visualise program theories within complex systems [25], and realist review methodology can be integrated with complex systems thinking in knowledge synthesis [26]; to date there has been a lack of combined application of systems approaches and realist methodology to program evaluation, in particular in the context of scale up. In addition, realist evaluation methodology has typically relied on intra-programme comparisons (i.e., comparisons across different groups involved in the same program) and testing the program theories of individual discrete programs. This has led to a debate surrounding the generalisability of CMO configurations [27], and questions regarding the extent to which there are ‘portable elements’ (e.g., generalisable aspects) of program theories [28] that can be applied across programs and/or processes. In the context of scaling up, identifying the portable elements (also referred to as ‘mid-range theories’) and how they can be commonly applied across a variety of areas [28], will improve our understanding of their influence on achieving specific outcomes when scaling interventions in similar contexts.

There is a gap in the literature surrounding the use of realist approaches to identify common elements of program theories across *multiple interventions* involving many stakeholders and contexts. There are also few studies that have attempted to understand and develop CMO configurations related to a *process* (i.e., scaling up) as opposed to a discrete intervention’s activities and outcomes. As such, how different parts of the system relate to one another when scaling interventions, in addition to the specific activities of the intervention that lead to certain outcomes, has yet to be explored. The purpose of the present study was to combine realist principles and systems analysis (causal loop diagrams to pictorially demonstrate the relationships between contexts, mechanisms and outcomes) to explore the drivers underpinning implementation outcomes at scale and how these differed by key academics, practitioners and policymakers involved. Instead of focussing on ‘what went on’ during scale up (i.e., barriers and facilitators experienced) and whether scale up led to a sustainable population health impact; a core aim was to ascertain whether generalisable CMOs existed, which could be applied to a process (scaling up) as opposed to primarily a program. Using CMO configurations, we hypothesise how scaling up processes occur in order to generalise about the mechanisms underpinning scale up.

## Methods

### Study design

The ‘Scaling Up InTErventions’ (‘SUITE’) project aimed to understand the mechanisms associated with successfully scaled up physical activity and nutrition interventions globally, including how mechanisms differ by scaling up context. Using data from Australian interventions included in the SUITE project, we conducted a mixed method study to identify and assess physical activity and nutrition interventions previously implemented at scale or planned for implementation at scale in Australia during 2010–18. We defined an ‘intervention’ as “a set of actions with a coherent objective to bring about change or produce identifiable outcomes” [29], but for the purposes of this study, included only researcher and non-researcher (e.g., community/government) led programs and initiatives, and excluded those described as a policy, strategy or government regulation. Included interventions were not required to have demonstrated effectiveness on health or behavioural outcomes. We define ‘successfully scaled up’ as those interventions that have achieved state or national roll-out via governments, and *not necessarily* having demonstrated any effectiveness on health or behavioural outcomes.

**Fig. 1:**
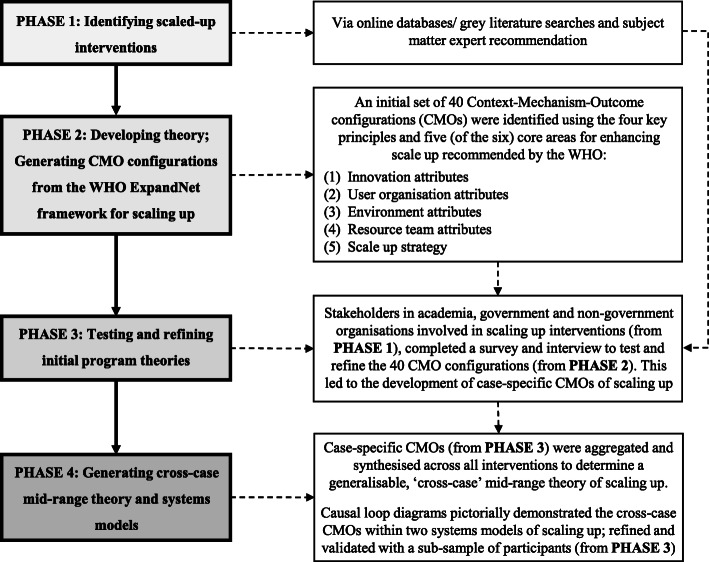
Study methodology

Participants included key stakeholders representing academic, government and non-government organisations (NGOs), involved in scale up. Drawing on a realist perspective, which adheres to RAMESES II reporting standards for realist evaluation [30], the study involved four distinct phases (Fig. 1). ***Phase 1*** identifying previously or currently scaled-up physical activity and/or nutrition interventions in Australia (2010–18); ***Phase 2*** generating initial program theories (in the form of CMOs) from the WHO ExpandNet framework for scaling up [9]; ***Phase 3*** testing and refining the program theories via online surveys and realist interviews, and; ***Phase 4*** generating a cross-case mid-range theory and systems models. For the purposes of this paper, CMO configurations are based on interview data and only descriptive data from online surveys is used. The design and methods of each phase are described below.

#### **Phase 1**: Identifying scaled-up physical activity and nutrition interventions

##### Inclusion criteria

**Fig. 2:**
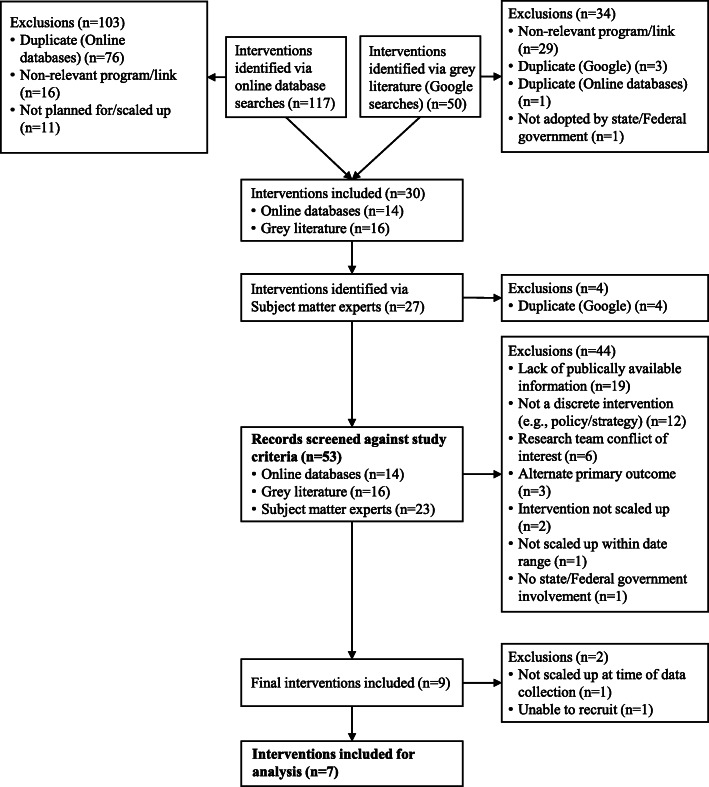
Intervention search results

Interventions were required to meet the following inclusion criteria: (i) implemented at a state, territory or national level during 2010–2017, or planned for implementation at a state, territory or national level during 2017–2018; (ii) rolled-out or planned for roll-out with a state or Federal government, as opposed to only via an industry or NGOs; (iii) a *primary* objective of improving physical activity and/or nutrition in accordance with the Australian Physical Activity Guidelines [31] and/or Australian Dietary Guidelines [32], respectively, and; (iv) publically available information on the intervention, scale up process and program lead contact details, to determine relevance to the study. As scale up is highly contextual, for the purposes of this paper, we only include interventions scaled up in Australia during 2010–18 to ensure we captured more recent scale up efforts and thus increase the potential generalisability of our findings as a result. Preliminary screening was conducted immediately after online and grey literature searches to remove duplicates, non-relevant programs/website links and those not planned or actually scaled up. Remaining interventions were then screened against the study inclusion criteria listed above. Figure 2 presents the search results.

##### Online database searches

Interventions were identified from the peer-reviewed literature (e.g. implementation/dissemination papers or protocol papers), using pre-specified search terms and online databases (e.g. EBSCO host) (databases and search strings are presented in Additional File 1). For consistency with previously published research and to ensure coverage of the appropriate literature, search strings were adapted from those used by Reis et al. (2016) [7] to the Australian context, and a research librarian was consulted during their development and testing. Database searches were conducted from 01/01/2010 to 05/02/2018 resulting in 117 interventions. Full articles were assessed in instances where intervention titles and abstracts provided insufficient information relating to the inclusion criteria. In total, 103 interventions were removed during preliminary screening, resulting in 14 interventions for assessment against the study criteria.

##### Grey literature searches

A grey-literature search was conducted via Google to capture any government or non-government led initiative that was not reported in the online peer-reviewed databases, (search strings are presented in Additional File 1). The search resulted in 42,100 hits, of which the first 50 results were preliminarily screened. In instances where the program link or publically available document provided insufficient information relating to the study inclusion criteria, further Google searches of the initiative were conducted (e.g., via the program’s website) to determine eligibility. Thirty-four interventions were removed during preliminary screening, resulting in 16 interventions for assessment against the study criteria.

##### Subject matter expert recommendation

To capture any additional potentially relevant interventions, an opportunity sample of 14 subject matter experts in an Australian University (Professors [*n* = 6] and senior academics [*n* = 8]), working in the design, implementation and/or scale up physical activity (*n* = 7) and nutrition (n = 7) interventions were approached and asked to identify specific examples of scaled up interventions within the Australian context. Experts were recruited to ensure equal representation of both nutrition and physical activity research, and identified 27 interventions, of which four were duplicates with the grey literature search. Twenty-three interventions were included for screening against the study criteria.

##### Final screening

Figure 2 presents a breakdown of the search results and reasons for exclusion. Fifty-three interventions were assessed against the study inclusion criteria by HK, SC, ML and JS (screened interventions listed in Additional File 2). Forty-four interventions were excluded, mostly due to a lack of publicly available information, as well as for a variety of other reasons, resulting in nine interventions which met the inclusion criteria. Two of the nine interventions were subsequently removed, as one was about to be scaled and we were not permitted to publish our data until after the program launch, and the other we were unable to recruit participants. Seven interventions were therefore included in the final analyses and they targeted improvements in nutrition (*n* = 1), physical activity (*n* = 1) or a combination of both (*n* = 5).

#### **Phase 2**: Developing theory: generating CMO configurations from the WHO ExpandNet framework for scaling up

The WHO ExpandNet framework for scaling up [9] provided the theoretical framework for this study and formed the basis for developing the initial program theories. The guide contains four key principles for enhancing scale up which relate to the intervention (i.e. components are perceived as new in a particular context), testing of the intervention (i.e. evidence-based), that scaling up involves deliberate efforts (i.e. a guided process as opposed to spontaneous diffusion) and, that it fosters program and policy development on a lasting basis (i.e. developing and establishing political support).

The guide incorporates six core areas for consideration during scale up: 1) **Innovation attributes** – for consistency, herein referred to as ‘**Intervention attributes’** - (e.g., features that increase the likelihood of an intervention being successfully transferred, such as credibility, relevance and compatibility); 2) **User organisation attributes** (e.g., organisational characteristics such as perceived need and implementation capacity); 3) **Environment attributes** (e.g., opportunities in the environment to minimise constraints or accelerate institutionalisation, such as the policy context and bureaucracy); 4) **Resource team attributes** (e.g., features that increase likelihood of attaining scale up goals, such as a unifying vision and effective leadership); 5) **Scale up strategy** (e.g., plans and actions necessary to establish intervention such as advocacy strategies, and monitoring and evaluation) and; 6) **Planning and management** (e.g., strategic monitoring and consistent attention to the scaling up process, and maintaining an appropriate balance among elements of scaling up system, such as recognising when trade-offs are necessary).

The four key principles and five of the six areas for consideration (excluding ‘planning and management’ based on relevance to the study aims) were used to identify an initial set of 40 CMOs for testing and refinement in Phase 3 (Additional File 3 presents application of the WHO ExpandNet framework to data collection measures). The 40 CMOs were derived by reviewing and mapping descriptive content within the WHO framework within an excel database against the six areas for consideration. For example, the WHO ExpandNet framework refers to the fact intervention attributes need to have “*relative advantage over existing practices so that potential users are convinced that the costs of implementation are counteracted by the benefits*” (Page 11), which contributed to the CMO “*Intervention was a new approach to existing efforts* [CONTEXT] - *Intervention provided an alternate ‘way’ of addressing problem (*i.e. *either* via *its components or strategy)* [MECHANISM RESOURCE] - *Intervention provided a greater advantage over previous efforts (*i.e. *integrated into existing practices or had never been done before)* [MECHANISM REASONING] - *Increased stakeholder buy-in and support for scale up, improved implementation and sustainability* [OUTCOME].

#### **Phase 3**: Testing and refining initial program theories

##### Participants and recruitment

Participants representing the seven scaled up interventions (identified in Phase 1) were grouped according to three stakeholder groups: 1) academia (University-based academics responsible for designing/testing the intervention), 2) government (policy-makers/civil servants involved in government adoption and/or implementation of the intervention), and 3) non-government (stakeholders in Non-Government Organisations [NGOs], industry or community-based organisations that had a significant role in the scale up process). Participants were recruited in two phases: (1) purposive sampling to identify individuals named in publications/reports associated with each intervention; and (2) a snowball sampling technique to identify additional individuals that had a significant role in scale up but may have been missed during purposive sampling.

##### Data collection procedure

All participants were invited via email to participate in an online survey (< 20 min) and follow-up telephone interview (< 1 h). Interviews were conducted in a realist ‘teacher–learner’ style [33] to explore participants’ experiences of scaling up. The teacher-learner style involves providing the interviewee with the program theory (i.e., concepts in the WHO ExpandNet framework for scaling up), which is subsequently commented on and refined accordingly. The roles of the teacher and learner are interchangeable throughout the interview in order to think through and understand the complexities involved [33]. This type of interviewing was integral to meet the aims of this study, as it was possible to move beyond simply exploring participants’ experiences of scaling. Instead, we were able to make inferences about the phenomena in question and re-test these inferences against existing theory or practice during interviews. Interviews identified important outcomes to test and refine the initial 40 CMO configurations, and were audio recorded for later transcription.

##### Measures

Twenty-five interview questions were used, derived from the WHO report ‘20 Questions for Developing a Scaling up Case Study’ [34], and the four key principles and five areas for consideration in the WHO ExpandNet framework for scaling up [9]. Example questions include: “*How did the intervention meet the goals or objectives of the different systems (e.g. health, education, policy priorities) that it was integrated into?*” (Intervention attributes); “*Were any strategies developed, either by the research team or stakeholders, to ensure the target settings had the capacity to actually implement what was required of them?*” (User organisation attributes); “*Did the political context at the time the intervention was ‘rolled-out’ affect your efforts or any resources that were needed for scale up?*” (Environment attributes); “*Was there a role for policy advocates? Were they used to promote the intervention within government or to make decisions on wider roll-out in general?*” (Resource team attributes); “W*hat were the advantages disadvantages to the approach [centralised or decentralised or both] taken in scaling up* (Scale up strategy), and; “*Was there a process to monitor and evaluate how the intervention was progressing?*” (Monitoring and evaluation). The key principles and areas for consideration in the WHO framework were used explicitly and systematically throughout the interview process, to represent an initial, rough program theory, which was then tested and refined by interviewees.

##### Analysis

Online survey data were analysed descriptively. Interview transcripts were transcribed verbatim for realist analysis. Three members of the research team (HK, SC, HD) read a sample of the interview transcripts, organising the data according to the initial program theories (CMOs), and creating new codes for instances of emergent CMO configurations. As participant experiences of scale up were anticipated to include both shared and unique perspectives, no minimum number of responses relating to a CMO was required for an emergent CMO to be accepted. Transcripts were coded by participant group (government, non-government and academia). Emergent CMOs were compared and discussed until consensus was reached between the three members of the research team, leading to a refined list of ‘case-specific’ CMOs. The case-specific CMOs related to each individual intervention were then synthesised across cases (by HK and SC). Synthesis involved identifying CMOs that contained similar or overlapping content and then combining these multiple CMOs to form one CMO that was representative for all the cases. Outcomes were then used to produce a set of CMOs reflecting participants’ experiences of the scaling up *process*.

#### **Phase 4**: Generating cross-case mid-range theory and systems models

To develop a generalisable interpretation of the mechanisms underpinning scale up, Phase 4 involved program theory refinement by determining which case-specific CMOs from interviews (Phase 3) offered the most robust and plausible explanation of the pattern of outcomes.

The case-specific CMOs were aggregated and synthesised across all cases according to common mechanisms, to inform a more generalisable ‘cross-case’ mid-range theory and hypotheses about the scaling up process. Due to participants’ integration of several concepts and/or emphasis of embedded concepts within the core areas, we combined ‘user organisation attributes’ and ‘resource team attributes’, and created a new area ‘monitoring and evaluation’, which was previously included within the ‘scale up strategy’ domain.

Systems analysis involves identifying components of a system, generating a mental model of the relationships between those components, and then depicting this model as a causal loop diagram [24]. For the purposes of this study, systems analysis was used to understand the relationships within individual CMOs underpinning the scale up process. In this paper, causal loop diagrams (herein referred to as ‘systems models’) provide a visualisation of the cross-case CMOs that were identified through interviews. Unlike other systems approaches, such as system dynamics, the pathways displayed in the systems models always begin with a scaling Context, end with a scaling Outcome, as a result of specific Mechanisms; and thus feedback loops are not depicted. The systems models were produced using Kumu relationship mapping software (2019) (Retrieved from https://kumu.io/). For refinement and model validation purposes, subject matter experts (a subsample of participants [*n* = 12] representing all three participant groups) provided feedback on the systems models via videoconferencing.

## Results

Of the seven scaled up interventions assessed, one targeted improvements in nutrition, one targeted physical activity, and five focused on a combination of both. Representing these interventions, 26 participants completed surveys (*n* = 4 government; *n* = 7 non-government; *n* = 15 academia), and of those, 19 participated in an interview (*n* = 3 government; *n* = 5 non-government; *n* = 11 academia). Tables 1 and 2 present descriptive information on participants and the seven scaled up interventions, respectively.

**Table 1:** Descriptive characteristics of interview participants

	Government (***n*** = 3)	Non-Government (***n*** = 5)	Academia (***n*** = 11)
**Age (range)**	35–39 years (1) 55–59 years (1) 45–49 years (1)	30–34 years (1) 40–44 years (1) 45–49 years (1) 50–54 years (1) 60+ years (1)	30–34 years (1) 40–44 years (2) 45–49 years (4) 60+ years (4)
**Sex**	Female (1) Male (2)	Female (4) Male (0) Prefer not to say (1)	Female (10) Male (1)
**Time in organisation**	< 1 year (1) 1–5 years (2)	< 1 year (1) 1–5 years (3) 6–10 years (1)	< 1 year (1) 1–5 years (4) 6–10 years (2) 11–15 years (1) 16–20 years (1) 21–25 years (1) > 25 years (1)
**Time involved with intervention**	> 1 < 6 years (1) ≥ 6 < 10 years (2)	< 1 year (1) ≥ 1 < 6 years (2) ≥ 6 < 10 years (1) Prefer not to say (1)	^a^ < 1 < 6 years (9) ≥ 6 < 10 years (1) ≥ 10 < 15 years (1)

^a^One participant reported time involvement in date range (years) only; therefore 12 months involvement per year was assumed

**Table 2:** Descriptive characteristics of scaled up interventions

Intervention name	Intervention type	Target outcome	Population and setting	Scale up time frame	Scale up level
**Stephanie Alexander Kitchen Garde **[35]	School-based food education program	Nutrition	Primary schools	2005- ongoing	National
**PEACH (Parenting Eating and Act for Child Health) **[36]	Community-based multi-component group educational sessions	PA & Nutrition	Families with overweight/obese children aged 5–11 years, Community settings	2013–2016 (QLD)	State (QLD)
**Munch and Move **[37]	Training and resources for early childhood educators	PA & Nutrition	Children aged 0–5 years, Early childhood education and care services	2013- ongoing	State (NSW)
**Live Lighter **[38]	Educational mass media campaign	PA & Nutrition	Adults, mass media and social media	2012–2015	State (WA, VIC, ACT & NT)
**Physical Activity 4 Everyone **[39]	Whole-school physical activity program	PA	Adolescents, Disadvantaged secondary schools	2017 – ongoing	State (NSW)
**OPAL (Obesity Prevention and Lifestyle) **[40]	Community development and social marketing	PA & Nutrition	Children through families, Community-based	2009–2017	State (SA)
**Go4Fun **[41]	After school obesity prevention program	PA & Nutrition	Children 7–13 above a healthy weight, Community settings	2009- ongoing	State (NSW)

Information in table relates only to scale up period for each intervention. *PA* Physical Activity, Australian states: *SA* South Australia, *QLD* Queensland, *NSW* New South Wales, *WA* Western Australia, *ACT* Australian Capital Territory, *NT* Northern Territory

### Mechanisms underpinning scale up: relationships with scaling up context and outcomes

Sixty-three pathways depicted relationships between 36 scale up contexts and 8 outcomes for successful scale up, linked via 53 commonly occurring mechanisms (relationships between mechanisms and outcomes described in Additional File 4). Of the 36 scale up contexts, 5 also had a role as mechanisms (e.g., innovation attributes [*n* = 4] such as the intervention was a ‘perceived priority/problem on stakeholder agenda’, and environment attributes [*n* = 1], the ‘impact of political instability’). The 53 mechanisms comprised both *resources* (e.g., materials to support implementation/funds) and *reasoning* (e.g., stakeholders’ responses to the context/situation). Whilst there were commonalities in participant experiences, mechanisms led to both intended and unintended consequences on scale up outcomes, which are described below. These differed based on the preceding context of scale up and participants’ perceptions of what was important and feasible when scaling (e.g., prioritising some aspects over others).

### Government participants’ perceived relationships in scale up

Overall, individuals working in government (*n* = 3) who were interviewed tended to have more of a systems perspective on what was required to achieve intervention embeddedness and sustainability at scale (e.g., discussion of the need for interaction and support for scaling across multiple systems involved), compared to non-government and academic participants. Four CMOs unique to this participant group emerged, corresponding to three of the five WHO core areas: ‘user organisation and resource team attributes’ (*n* = 2), ‘environment attributes (policy context)’ (*n* = 1) and ‘scale up strategy’ (*n* = 1). Enhanced long-term sustainability was attributed to early involvement of policy entrepreneurs and champions during intervention design and testing. Key mechanisms were the early identification of resources and strategies at multiple levels of government, both within and outside of the health system. This meant government buy-in and support, and thus politically well-connected advocates, were able to champion the timing of scale up. In relation to the policy context, sustainability was perceived to increase due to the scale up approach leveraging opportune political moments, which was largely achievable if scaling was designed to be responsive to real-world conditions and thus changes in the political environment. Conversely, a lack of understanding of the strengths/weaknesses in the delivery setting was linked to both potential reductions in uptake and effective implementation at scale, yet also enhanced implementation. Implementation could be enhanced, despite a lack of prior understanding of the delivery setting, due to the fact that resources (e.g. training) or incentives (e.g. supportive policies) were retrospectively integrated, which minimised the impact of weakness as roll-out takes place. A key mechanism in this instance was the avoidance of putting in ‘parallel’ processes (i.e., duplicate or conflicting processes that already existed in the delivery setting), irrespective of whether these processes were identified prospectively or retrospectively. An understanding of the costs of going to scale prior to roll out associated with increased intervention implementation and sustainability, but critically, these resources needed to be explicit and from diverse sources.

### Non-government participants’ perceived relationships in scale up

Participants representing NGOs (*n* = 5) had a broadly consistent understanding of the mechanisms underpinning scale up as with government participants. Nine CMOs unique to this participant group were developed, corresponding to four of the five WHO core areas: ‘intervention attributes’ (*n* = 5), ‘environment attributes (policy context)’ (*n* = 1), ‘scale up strategy’ (*n* = 2) and ‘monitoring and evaluation’ (*n* = 1). Participants described increased stakeholder buy-in and support for scale up as associated with evidence, but also pointed out that this evidence need not relate to the target purpose of the intervention. Rather, legitimacy of the evidence base was perceived based on ‘value’ of the intervention and evidence that may be indirectly related (e.g. evidence for the intervention strategy or underpinning model more broadly, but not necessarily for the outcome of interest). The intervention was still perceived as credible and politically favourable, despite not providing direct evidence for impact, as it provided a new/alternate strategy than previous approaches. These perceptions were a key mechanism for increased legitimacy in the community and among stakeholders, and subsequent scale up. In addition, when stakeholder expectations and perceptions of important/persuasive evidence were taken into consideration over time, increased stakeholder confidence and subsequent buy-in to support the intervention was achieved. Tailoring data collection to collect evidence perceived as credible among stakeholders was a key mechanism for buy-in, despite that this need not necessarily lead to practice/policy impact.

Conversely, interventions that were not designed for scale up when originally tested, meant retrospective resource (physical and fiscal) development was required (e.g., change agent role moving from centralised position to become included in end user organisation). As a result, reactive actions to support the intervention and implementation process was perceived more appropriate for real-world settings, leading to potentially greater sustainment at scale. Whilst interventions may lead to outcomes relevant to multiple systems (i.e., health and education systems), an overemphasis on health outcomes compared to others, lead to a lack of system-level intervention embeddedness at scale. The key mechanism in this instance was the lack of alignment with the goals of the system(s) that the intervention is linked to and thus not on the agenda of multi-sector stakeholders.

In terms of environmental attributes (political context), only participants from NGOs referred to a thorough understanding of government/political structures as an enabler to strategically position key advocates to gain political support for scale up. This understanding was perceived to be effective as it resulted in the resources (e.g. time and funds) for stakeholder engagement and embedding the intervention in practice were planned for. A ‘bipartisan’ approach to strategic engagement ensured key government stakeholders supporting the intervention were present, and the intervention retained high awareness and value among key political actors. As a result, political support for scaling was initially maintained.

Regarding the scale up strategy, only NGO participants described diversifying support/resources for implementation from within and outside of government (e.g., philanthropic, corporate) to enable ‘robustness’ during unstable political climates such as changes in government and funding. Planning resources so the intervention remained ‘on the agenda’ was linked to sustainable commitment to implementation. Participants described how a lack of embedded monitoring and evaluation within the implementation/scale up process from the outset, meant ‘visual accounts’ of implementation (e.g., presence of intervention components during site visits) were used to infer intervention impact. Measures were retrospectively put in place in response to stakeholder requirements for monitoring data, however, the absence of formal measures from the outset undermined evidence of impact and limited practice/policy impact. Perceived lack of understanding and value placed on formal evaluation measures compared to more powerful/important subjective ‘visual accounts’ of impact, was a key mechanism underpinning this scale up outcome.

### Academic participants’ perceived relationships in scale up

Overwhelmingly, compared to other participant groups, academics (*n* = 11) emphasised monitoring and evaluation and the role of evidence as a crucial mechanism in the decision to scale interventions and sustain their implementation. Twelve unique CMO configurations emerged among this group, corresponding to four of the five WHO core areas: ‘intervention attributes’ (*n* = 5), ‘environment attributes (policy context)’ (*n* = 1), ‘scale up strategy’ (*n* = 3) and ‘monitoring and evaluation’ (*n* = 3). In general, participants had less of a systems perspective and understanding of what was required for system embeddedness at scale. Academics described the context of an intervention being evidence-based, but based on testing in a different context (e.g., tested in another State) or aspects of the intervention being evidenced-based (e.g., underlying concepts) but tested in a controlled trial as opposed to a real-world setting. Irrespective of the evidence-base context, participants reported that an intervention was perceived as credible and more likely to solve the problem, and thus generated increased stakeholder buy-in and support for scale up. According to the academics, a direct evidence base was not required for stakeholder buy-in; however, stakeholder expectations of important/persuasive evidence were taken into consideration, potentially leading to less evidence for impact on target outcomes due to the focus of the measures used. Mechanisms underpinning this related to the conflict between academic’s preferences for evaluation measures and stakeholder perceptions of persuasive evidence.

There was a direct relationship between the environmental context (policy) and a lack of evidence for the potential impact of an intervention on the target outcomes. Political favourability meant sustained political funding and support was observed, whereas, a lack of understanding of complex government/political structures lowered political support for scale up. This was perceived as a result of unanticipated resources required during scaling up, and low awareness and value placed on the intervention among key political advocates. In relation to scale up strategies, spontaneous rollout as a response to political funding and need to meet targets, meant interventions were omitted from a long-term sustainability agenda at all levels. Lack of preparation and lead in time meant the focus of scale up was on achieving scale up targets (i.e., reach). Such focus meant the intervention was perceived as an additional ‘extra’ to already committed program agendas, as opposed to ensuring quality of the scale up process and achieving embeddedness.

### Systems models of scaling up

**Fig. 3:**
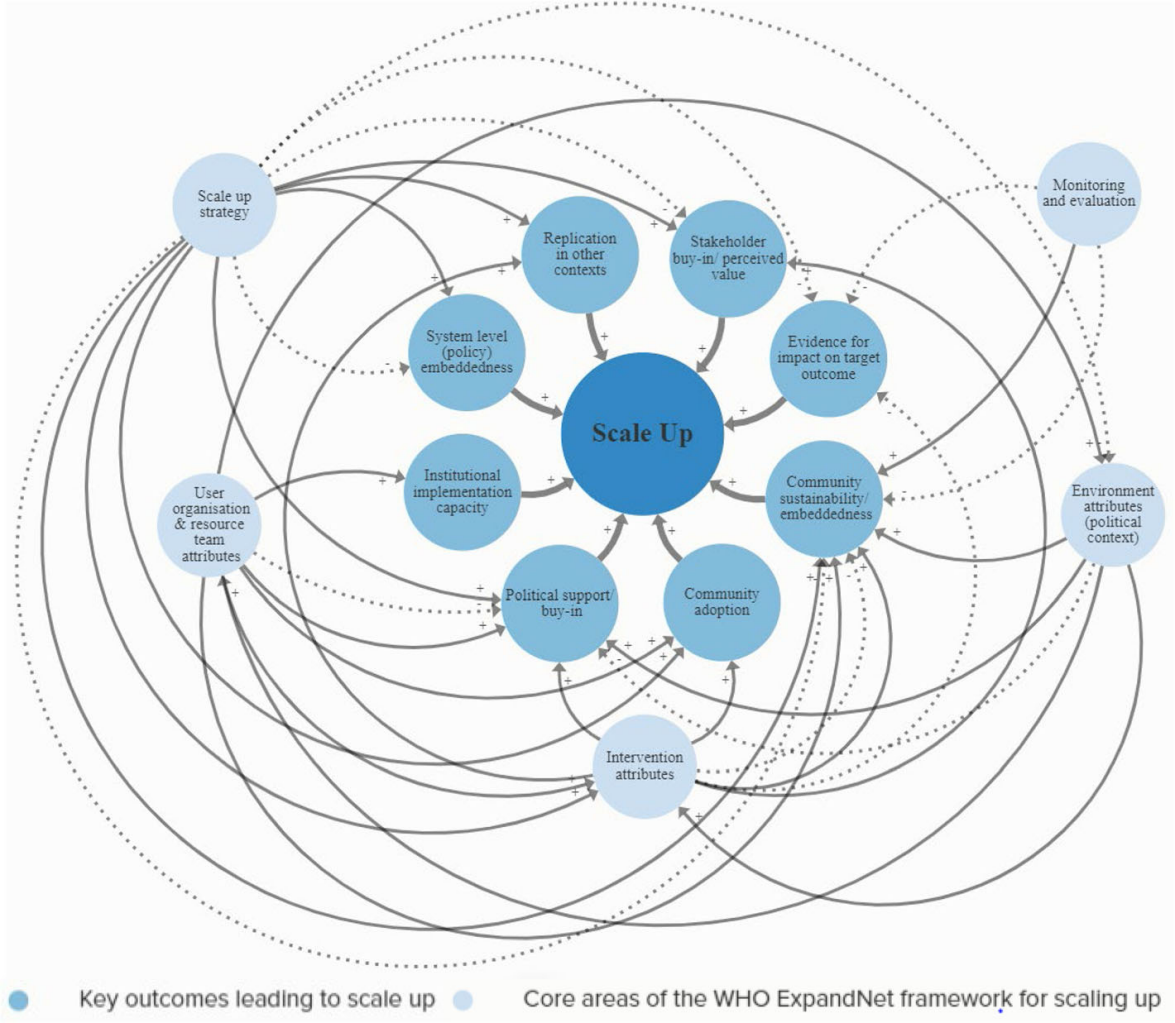
Systems Model of Scaling Up. Solid black arrows (positive relationship), dotted black arrows (negative relationship)

**Fig. 4:**
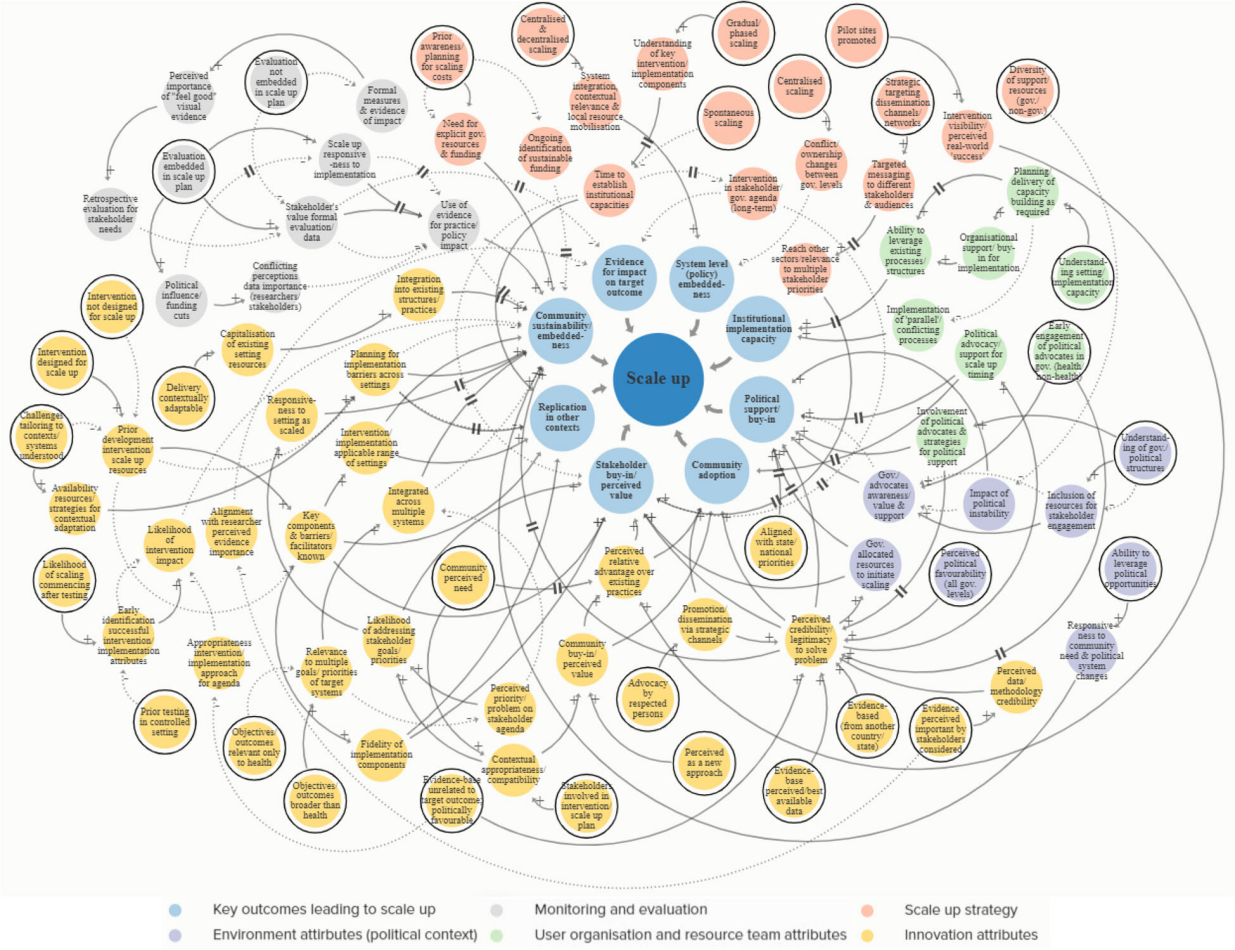
Complex Systems Model of Scaling Up. Colours correspond to core areas of the WHO ExpandNet framework. Solid black arrows (positive relationship), dotted black arrows (negative relationship). Black rings = ‘Context’ variables. **||** symbol = delay

The cross-case CMOs and interrelations generated from interview data during Phase 4 were integrated into two systems models of scale up. Figure 3 presents a *Systems Model of Scaling Up,* which depicts the relationship between eight major outcomes underpinning scale up and the five key areas in the WHO framework. Figure 4 presents an expanded version of Fig. 3, a *Complex Systems Model of Scaling Up*, which illustrates, in detail, 63 different CMO configurations (pathways) leading to successful scale up. Variables in the *Complex Systems Model* were colour coded according to the five core WHO framework areas, with delay symbols indicating the dynamics (time lags) between variables. Those variables highlighted with black rings indicate a ‘Context’ for scaling. Member checking confirmed the relationships depicted in the systems models, and led to refinement of visual and descriptive aspects for greater clarity (e.g., removing coloured lines that misleadingly implied a greater ‘importance’ of some areas over others, and providing in-text definitions of some terms used).

The five key areas of the WHO framework were all linked to the eight key outcomes for successful scaling, however the number, length and complexity of the CMO pathways varied greatly. The greatest number of pathways corresponded to **intervention attributes** (*n* = 29), most of which led to community sustainability/embeddedness (*n* = 11), followed by stakeholder buy-in/perceived value (*n* = 8), replication in other contexts (*n* = 4), community adoption (*n* = 4), political buy-in/support (*n* = 1) and evidence for impact on the target outcome (*n* = 1). **Scale up strategy** pathways (*n* = 13) led to stakeholder buy-in/perceived value (*n* = 4), community sustainability/embeddedness (*n* = 3), systems level (policy) embeddedness (*n* = 2), replication in other contexts (*n* = 1), evidence for impact on target outcome (*n* = 1), community adoption (*n* = 1), and political support/buy-in (*n* = 1). **User organisation and resource team attributes** pathways (*n* = 8) led to institutional implementation capacity (*n* = 3), political support/buy-in (*n* = 3), community sustainability/embeddedness (*n* = 1) and community adoption (*n* = 1). **Environment attributes** (political context) pathways (*n* = 7) led to political support/buy-in (*n* = 5) and community sustainability/embeddedness (*n* = 2). **Monitoring and evaluation** pathways (*n* = 6) led to community sustainability/embeddedness (*n* = 5) and only one was linked to evidence for impact on the target outcome.

## Discussion

To our knowledge, this paper provides the first evidence for mechanisms underpinning outcomes required for the successful scale up of a selection of state or nationally delivered physical activity and nutrition interventions in Australia. This paper also demonstrates a novel approach to determining generalisable relationships when scaling up interventions with governments, by combining a theory-driven realist and complex systems approach, within a multiple cross-case comparison design. Our systems models of scale up provide a visual tool for policy-makers, practitioners and academics wishing to plan and evaluate future scaling efforts. Findings from this study address a major gap in the scale up literature. Our outcomes provide theoretically generalisable relationships between scaling up contexts and subsequent outcomes, highlighting commonly occurring mechanisms that could potentially be leveraged during future scale up efforts, to influence population impact. Findings will advance academic, practitioner and policymaker knowledge of possible actions and leverage points to achieve population impact of interventions at scale.

Consistent with previous research that has categorised the varied pathways of scale up [49] and suggested non-linearity of the process [42], our findings explicitly illustrate the inherent complexity of scale up, including the many interrelations between and within relevant scale up domains. The findings thus demonstrate that achieving intervention implementation at scale is dependent on a complex set of interacting variables. Although the eight core outcomes we identified as key to successful scale up were consistent with the WHO framework for scaling up [9], their perceived importance, role and relationship to other factors when scaling up differed greatly. Unsurprisingly, the most commonly occurring and consistent relationships were those related to the WHO framework domain ‘intervention attributes’. More than three times the number of mechanisms and more than double the number of pathways were identified in this domain, compared to the four other areas of the WHO framework. Emphasis on the intervention as the focal point of scaling is to be expected given that the intervention is the primary entity that is being replicated or expanded, and may be what individuals involved in scaling have greatest control and influence over.

Although the WHO scale up framework characterises scaling up as an “open system with interrelated elements” [9], we found emphasis on the framework domains differed greatly. For example, environment attributes (policy context) and user organisation/resource team attributes had the fewest number of variables. Outcomes ‘institutional implementation capacity’ and ‘system level (policy) embeddedness’ had the fewest number of CMO pathways. Consistent with previous research, which identified relationships with local and national stakeholders was perceived integral to scale up success [43]; we found community sustainability/embeddedness and stakeholder buy-in/perceived value elicited the greatest number of pathways. These findings may be interpreted in several ways. Firstly, the delivery and implementation context, including those supporting implementation and scale up (e.g., the resource team), and system level institutionalisation may have a far less overt role and perceived impact on the outcomes and process of scaling up. Community sustainability/embeddedness and stakeholder buy-in/perceived value may have (or is perceived to have) a greater and more complex role in scaling, and thus there are more mechanisms and contexts involved. It could also reflect the focus of knowledge and understanding of scaling in public health, which typically focuses on the reach of interventions over and above systems-related factors [42]. Although the role of health system strengthening is increasingly recognised when scaling up international health interventions [44], our findings could also reflect the lack of mainstream recognition and understanding of the importance of the implementation capacity of the delivery setting, and system-level integration of factors when scaling in physical activity and nutrition.

Previous research suggests that evidence for intervention effectiveness in some areas is a prerequisite for scaling up [45] and yet our study showed that this need not be the case. Only three of the five WHO framework domains (intervention attributes, scale up strategy, and monitoring and evaluation), were linked to ‘evidence for impact’ as a target outcome, eliciting only one CMO pathway each. Further, of the six CMO pathways related to ‘monitoring and evaluation’, only one pathway, discussed by academics, was linked to evidence for impact. This suggests that intervention evidence may play a much smaller role in the decision making process to commence and sustain scaling of interventions than the other outcome variables. It could also highlight that intervention evidence need not be sufficient at meeting the needs of decision makers when determining which interventions are ‘fit for purpose’, as intervention designs may be inappropriate for translation into real-world settings or at scale [14].

Research has also suggested that the potential for effective scaling includes, but is not limited to, its reach, adoption, costs when delivered at scale and acceptability/fit within the local context [9]. Again, we found inconsistent evidence to support this. Whilst the presence of these factors in our study were linked to increased likelihood of scale up in some circumstances, their presence was not *imperative* for an intervention to be rolled out state-wide or nationally. In fact, we found no difference in the CMO pathways for the likelihood of an intervention being scaled up based on direct evidence of effectiveness versus perceived potential intervention impact. This finding may contribute to explaining the lack of evidenced-based physical activity interventions that have been successfully scaled [7]. Intervention advocacy by respected persons in the community or government could result from three different sources of evidence: 1) *indirect*/*related evidence* of impact (e.g., evidence from another country or based on best available data that need not relate to the target outcome); 2) *perceived evidence* of impact and *perceived* credibility (e.g., ‘feel good’ visual observations of participant enjoyment as opposed to actual behaviour change), and; 3) *legitimacy of the evidence* (based on the extent the intervention was valued, e.g., evidence related to the intervention strategy or underpinning model more broadly, but not necessarily for the outcome of interest); all led to.

As has been shown previously [12], we found embedded monitoring and evaluation meant scale up was responsive to implementation and potentially enhanced. However, we found that evaluation data were perceived as more likely to be used when governments considered it as persuasive to address the issue at hand. Neither the quality nor quantity of evidence was imperative for informing physical activity and nutrition policy and practice, rather, whether the evidence was ‘fit for purpose’. For example, the intervention may have had a demonstrable evidence base, but it could be used to address a public health issue that it was not originally intended for (e.g., designed to increase cooking skills and knowledge, but implemented as a strategy to reduce population obesity). Whilst it has been shown that inadequate integration of research into scale up efforts can impede the success of scale up, we found having an evaluation embedded in the scale up plan (context) could lead to both an increase and decrease in the likelihood of community sustainability/embeddedness (outcome), depending on stakeholder perceived value and understanding of formal evaluation data (mechanism). Stakeholder buy-in and confidence of the intervention was enhanced when their expectations and perceptions of persuasive evidence were taken into account. This was in spite of there being a perceived tension between academics and stakeholders over the importance, purpose, design, conduct and use of scale up evaluation data. Whilst this highlights the importance of stakeholder involvement in the design of interventions to ensure outcomes meet the needs of those involved [14], it also highlights that what is regarded to be quality evidence synthesis and translation can be value-laden. Despite being potentially unrelated to any practice/policy impact, tailored data collection was perceived as credible among stakeholders, and was key to subsequent buy-in. However, this raises questions regarding the legitimacy of evidence generation and value-driven evidence use. These findings highlight important opportunities to use program theory for intervention development, and the use of participatory research designs to strengthen and embed mutually agreed monitoring and evaluation frameworks from the outset. Participatory research can facilitate exploration of stakeholder perceptions of evidence credibility, prior to evidence generation, and is critical in planning for scale up [14].

Political alignment was the cornerstone of effective scaling in the interventions included in this study, with scale up encompassing much more than just ‘implementation of the intervention at scale’. Being reactive to unpredictable and emerging issues in the implementation and policy context was also linked to increased sustainability at scale. Research into scale up from a complex adaptive systems perspective supports the notion of preparing for unpredictatbility, to enable continual adaptation to stakehlder needs [46]. Likewise, beyond the benefits of a research teams’ extensive knowledge of and experience with stakeholders involvd in scaling processes, and what is required for ‘implementation at scale’ [43]; there was still contextual variability of the systems involved. For example, we found that irrespective of similarities in the scale up context (e.g., a prior understanding of government/political structures), both intended and unintended scale up outcomes occurred (e.g., political support for the intervention could increase or decrease as scale up was underway). This type of variable and dynamic relationship has important implications for future approaches towards planning and assessing potential scalability of interventions, in particular how we anticipate the likelihood of system-wide institutionalisation [14]. The bi-directional and dynamic relationships we identified between contexts, mechanism and outcomes may contribute to explaining why there is such huge variance in the success of scale up efforts. Yet, if we consider current tools and guides aimed at practitioners, policy makers and/or academics for determining intervention scalability, the rhetoric is centred on a linear ‘context-to-outcome’ relationship. Establishment of an ideal context for scaling (e.g., demonstrable intervention effectiveness) is described as increasing the likelihood of achieving an ideal outcome (e.g., increased community reach and adoption), and yet the role of mechanisms has largely been ignored.

Whilst planning for implementation and scale up is recommended for improved scalability of population health interventions [7, 14], implementation models are typically underused in the planning stages of real-world physical activity interventions [47]. We identified 53 different mechanisms leading to successful scale up. This is almost double the number of potential contexts for scaling and more than six times the number of key outcomes we found in this study; some of which were responsive to time and effect as a result of ‘delays’. Whilst tools and resources do provide a beneficial and systematic way of ascertaining if certain parameters exist prior to scaling, including what influence they may have; their assumed, linear, context-to-outcome relationship potentially oversimplifies what leads to an intervention being adopted and widely implemented by governments. For complex public health issues [48], a lack of emphasis on the non-linear interactions between scaling contexts, mechanisms and outcomes in current tools and resources to assess intervention scalability; may limit their effectiveness in practice. Whilst our findings reiterate the importance and role of factors within the five domains of the WHO ExpandNet framework for scaling up, our results build on this tool by illustrating the different interaction between the domains and the relative importance placed on the core areas by those involved in scaling interventions. As such, we anticipate that our systems models will extend existing resources dedicated to the planning, implementation and evaluation of scale up in consideration of systems, and can be used for internal and external advocacy, to generate funding, and as a tool/checklist to illustrate the dynamic and complex adaptive nature of scaling up.

We believe that the lack of current evidence in the broader literature for mechanisms leading to successful scale up, contributes to our lack of understanding of how scaling occurs, why some interventions are successfully scaled over others, and how we might predict and improve future scaling approaches. It may have also misdirected our focus on targeting those aspects which are more easily influenced by academics, practitioners and policymakers, such as intervention attributes and a focus on outcomes at scale, when other more distal factors such as perceived political favourability and credibility of the evidence has received less focus in planning for scale up.

### Strengths and limitations

A key strength of this study is the integration of multiple interventions and scale up contexts to develop potentially generalisable mechanisms for future intervention. Realist informed evaluation enabled a greater level of insight into the complexities of scale up, advancing our knowledge of possible actions and leverage points to influence contexts and activate mechanisms, to enhance both academic, practitioner and policymaker efforts to achieve population impact in complex environments and at scale. Recruiting participants working in government, community-based organisations and in research enabled a richer understanding of the complexities of scaling. By validating our systems models with subject matter experts, we have greater confidence in the accuracy and generalisability of our results.

This study is not without limitations. Firstly, our inclusion criteria required that interventions were described in a sufficient level of detail to determine eligibility (e.g., clear description of the primary objective). There is the possibility that some relevant interventions may have been excluded due to lack of publically available information. Lack of available data on scaled programs is not uncommon in public health research; Reis et al. identified an additional 56 scaled up physical activity interventions globally via expert consensus to those identified in their literature searches (*n* = 16) [7]. Whilst we cannot rule out the possibility that some scaled interventions may have been missed, to minimise the potential for this we undertook a three phase search strategy involving peer-reviewed and grey literature searches, followed by expert consultation. Nonetheless, the lack information on scaled interventions in the public domain does raise broader questions regarding the transparency of government-led programs and the potential misalignment of real-world innovations with stringent research parameters. Secondly, the search strings used in this study were adapted from those used by Reis et al. [7], which we developed and tested in consultation with a research librarian. Although this strategy helped promote consistency with the broader literature in the field, there is always the potential that some relevant publications/interventions may have been missed due to differences in terminology used.

## Conclusion

This paper provides important new evidence for the complex, non-linear process of scaling up interventions at a state or national level. Our findings challenge what is currently considered as prerequisites for effective scaling and criteria for intervention scalability, suggesting other conditions may be imperative to determine the likelihood of intervention adoption and implementation by governments at a state or national level. Scale up frameworks, assessment tools and guides for planning and evaluation may need to move beyond a linear context-to-outcome depiction of the scale up process, to better incorporate the complexity and impact of mechanisms on the scaling process. Future implementation and scale up may be enhanced by approaches that plan for complexity and adopt iterative strategies that encourage adaptation. Future research which disaggregates mechanisms further, to understand why an evidence-based intervention may fail to achieve successful scale up or the anticipated benefits in different contexts, would enhance how we implement strategies when scaling at a state or national level. The models developed in this study could also be used to support the development of computational system dynamics models, which in turn could be used to identify different leverage points and simulate the likely effectiveness of different interventions at scale. Greater awareness and consideration of the complex interactions between the perceptions, worldviews, values, goals and agendas of those involved in scaling interventions may be necessary to increase the potential adoption and ongoing implementation of evidence-based interventions at a population level.

## Supplementary Information

**Additional file 1.** Online database search strategy. Description of the online database search strategy.

**Additional file 2.** Table of Interventions for Screening. Descriptive table of 53 interventions screened for inclusion in the study.

**Additional file 3.** Application of the WHO framework. Table of WHO ExpandNet framework principles and core areas, and their application to the qualitative (interview) and quantitative (survey) data collected.

**Additional file 4.** Mechanisms underpinning scale up. Table listing the 53 commonly occurring mechanisms underpinning scale up, and their relationship to the eight key outcomes.

## Data Availability

The datasets used and/or analyzed during the current study are available from the corresponding author on reasonable request.

## Abbreviations

WHO: World Health Organization
PA: Physical Activity
CMO: Context-Mechanism-Outcome
RCT: Randomised Controlled Trial
SA: South Australia
QLD: Queensland
NSW: New South Wales
WA: Western Australia
ACT: Australian Capital Territory
NT: Northern Territory
