# Supplementary material for: Mechanisms of scaling up: combining a realist perspective and systems analysis to understand successfully scaled interventions
Source: Int J Behav Nutr Phys Act. 2021 Mar 22;18:42. doi: 10.1186/s12966-021-01103-0 (PMC7986035; doi:10.1186/s12966-021-01103-0)
Supplement: Supplementary file 1 — Additional file 1. Online database search strategy. Description of the online database search strategy. [file 12966_2021_1103_MOESM1_ESM.docx]

**Additional File 1. Online databases and search strings**

*Online database search*

via EBSCO host, Academic search complete, Ageline, CINAHL complete, Education Source, Environment Complete, ERIC, Community and mass media complete, Global Health, Health policy ref centre, Medline complete, Political science complete, and Sport Discuss. The search conducted on the 2^nd^ of February, 2018 returned 117 hits using the following search string; (("physical activ*" OR diet* OR nutrition*) AND (intervention* OR program* OR initiative*) ) ) AND AB Australia* AND ( (national OR state OR Victoria OR “New South Wales” OR “Northern territory” OR “Australian Capital Territory” OR “Western Australia” OR “South Australia” OR Tasmania OR Queensland ) N5 implement*) ).

*Grey literature search*

An advanced Google search using only the ‘.gov.au’ domain, the search string ("physical activity" OR diet OR nutrition AROUND 2 implement) (intervention OR program OR initiative) (national OR state OR Victoria OR “New South Wales” OR “Northern territory” OR “Australian Capital Territory” OR “Western Australia” OR “South Australia” OR Tasmania OR Queensland) returned 42,100 hits on the 5^th^ of February 2018.
