# Supplementary material for: Mechanisms of scaling up: combining a realist perspective and systems analysis to understand successfully scaled interventions
Source: Int J Behav Nutr Phys Act. 2021 Mar 22;18:42. doi: 10.1186/s12966-021-01103-0 (PMC7986035; doi:10.1186/s12966-021-01103-0)
Supplement: Supplementary file 2 — Additional file 2. Table of Interventions for Screening. Descriptive table of 53 interventions screened for inclusion in the study. [file 12966_2021_1103_MOESM2_ESM.docx]

**Additional File 2. Screened interventions**

| **#** | **Intervention name** | **Setting** | **Target outcome** | **Source (n=53)** | | | **Reason for exclusion** |
| --- | --- | --- | --- | --- | --- | --- | --- |
|  |  |  |  | *Peer-reviewed literature (n=14)* | *Grey literature (Google search) (n=17)* | *Subject matter experts*  *(n=28)* |  |
| **1** | Go4Fun (7) | After School Program | PA & Nutrition |  |  | X | INCLUDED |
| **2** | LiveLighter (6) | Community | PA & Nutrition |  |  | X | INCLUDED |
| **3** | Munch and Move (5) | Early Childhood education & care services | PA & Nutrition |  | X |  | INCLUDED |
| **4** | OPAL (Obesity Prevention and Lifestyle) (2) | Community | PA & Nutrition |  |  | X | INCLUDED |
| **5** | PEACH (Parenting Eating and Activity for Child Health) (1) | Community | PA & Nutrition |  |  | X | INCLUDED |
| **6** | Physical Activity 4 Everyone (PA4E1) (3) | Schools | PA |  | X | X | INCLUDED |
| **7** | Stephanie Alexander Kitchen Garden program (4) | Schools | Nutrition |  |  | X | INCLUDED |
| **8** | Healthy Workers Initiative (46) | Workplaces | PA & Nutrition | X |  |  | *INCLUDED after screening* – Excluded as unable to recruit |
| **9** | Learn your fruit and veg (Jamie Oliver) (8) | Community | Nutrition |  |  | X | *INCLUDED after screening* -Excluded as not scaled up at point of data collection |
| **10** | Active for life (22) | Community | PA |  |  | X | Not a discrete intervention (e.g., policy/strategy) |
| **11** | Applying a performance monitoring framework to increase reach and adoption of children's healthy eating and physical activity programs (47) | Early childhood education and care, and primary schools | PA & Nutrition | X |  |  | Not a discrete intervention (e.g., policy/strategy) |
| **12** | Australian National Breastfeeding Strategy 2010-2015 (44) | Stakeholders | Nutrition | X |  |  | Not a discrete intervention (e.g., policy/strategy) |
| **13** | Be Active (18) | Local council | PA |  | X |  | No state/Federal government involvement |
| **14** | Beat it (20) | Structured group sessions | PA & Nutrition |  | X |  | Lack of publically available information |
| **15** | CAFÉ (41) | Schools | Nutrition | X |  |  | Intervention not scaled up |
| **16** | Crunch&Sip® (24) | Schools | Nutrition |  | X | X | Lack of publically available information |
| **17** | Finish with the Right Stuff (35) | Community sport | Nutrition |  | X |  | Lack of publically available information |
| **18** | Food Sensations® for Adults (38) | Community | Nutrition |  |  | X | Lack of publically available information |
| **19** | Foodcents (40) | Community | Nutrition |  |  | X | Alternate primary outcome |
| **20** | Get Healthy Information and Coaching Service® (36) | Telephone information and support service | PA & Nutrition |  |  | X | Lack of publically available information |
| **21** | Girls make your move (30) | Community | PA & Nutrition |  |  | X | Lack of publically available information |
| **22** | Go for your life (42) | Primary and Pre-Schools, Early Childhood services | Nutrition | X |  |  | Lack of publically available information |
| **23** | Government regulation to promote healthy food environments (49) | Policy | Nutrition | X |  |  | Not a discrete intervention (e.g., policy/strategy) |
| **24** | Healthy canteen policy (10) | Schools | Nutrition | X |  |  | Not a discrete intervention (e.g., policy/strategy) |
| **25** | Heart Foundation Tick Program (19) | Community | Nutrition |  |  | X | Lack of publically available information |
| **26** | Heart Foundation Walking (17) | Community | PA |  | X | X | Research team conflict of interest |
| **27** | Heart Foundation Heartmoves (25) | Gym, run by fitness experts | PA |  | X | X | Lack of publically available information |
| **28** | Healthy Living after Cancer (11) | Telephone information and support service | PA & Nutrition | X |  |  | Research team conflict of interest |
| **29** | Healthy Dads Healthy Kids (26) | Community | PA |  |  | X | Intervention not scaled up at point of data collection |
| **30** | Healthy Together Victoria (28) | Early childhood services, schools and workplaces | PA & Nutrition |  |  | X | Not a discrete intervention (e.g., policy/strategy) |
| **31** | Healthy food partnership (33) | Government, Public Health Sector, Food Industry | Nutrition |  |  | X | Not a discrete intervention (e.g., policy/strategy) |
| **32** | Increasing the provision of preventive care by community healthcare services: a stepped wedge implementation trial (45) | Community | PA & Nutrition | X |  |  | Alternate primary outcome |
| **33** | iPLAY (21) | Schools | PA |  |  | X | Research team conflict of interest |
| **34** | Jamie's Ministry of Food (29) | Community | Nutrition |  |  | X | Lack of publically available information |
| **35** | Life! (9) | Structured group sessions | PA & Nutrition | X | X |  | Not scaled up as a discrete intervention – scaled up as part of a collection of interventions (Healthy Together Victoria) |
| **36** | Lift for life (53) | Community | PA |  | X |  | Lack of publically available information |
| **37** | Live Life Well @ School (34) | Schools | PA & Nutrition |  | X |  | Lack of publically available information |
| **38** | Melbourne InFANT Program (43) | Community | PA & Nutrition | X |  |  | Research team conflict of interest |
| **39** | National Healthy School Canteens Project (31) | Schools | Nutrition |  |  | X | Lack of publically available information |
| **40** | National strategy for food security in remote Indigenous communities (50) | Policy | NUT |  | X |  | Not a discrete intervention (e.g., policy/strategy) |
| **41** | National Partnership Agreement on Preventive Health (NPAPH) (15) | State monitoring of scaled up programs | PA & Nutrition | X |  |  | Not a discrete intervention (e.g., policy/strategy) |
| **42** | NSW Healthy Children Initiative (HCI) (52) | Community | PA & Nutrition |  | X |  | Not a discrete intervention (e.g., policy/strategy) |
| **43** | Queensland Country Womens Association Country Kitchens (37) | Community | Nutrition |  |  | X | Lack of publically available information |
| **44** | SecondBite (27) | Community | Nutrition |  |  | X | Alternate primary outcome |
| **45** | Smart choices (48) | Schools | Nutrition | X |  |  | Not scaled up within date range |
| **46** | Smart moves (16) | Schools | PA | X |  |  | Lack of publically available information |
| **47** | Swap it (23) | Schools | Nutrition |  | X | X | Lack of publically available information |
| **48** | The Heal™ Program (39) | Community | PA & Nutrition |  |  | X | Lack of publically available information |
| **49** | The Health Star Rating (32) | Government, industry, public health, and consumer organisations | Nutrition |  |  | X | Not a discrete intervention (e.g., policy/strategy) |
| **50** | Walk Safely to School (14) | Schools | PA |  |  | X | Lack of publically available information |
| **51** | Walk to School (formally walking school bus) (13) | Community/schools | PA |  | X |  | Research team conflict of interest |
| **52** | YMCA Schools’ Breakfast Program (51) | Schools | Nutrition |  | X |  | Lack of publically available information |
| **53** | 10,000 Steps (12) | Whole of community | PA |  | X |  | Research team conflict of interest |

PA – Physical Activity, RCT – Randomised Controlled Trial, NSW – New South Wales, YMCA - Young Mens' Christian Association

**References**

1. Moores CJ, Miller J, Perry RA, Chan L, Daniels LA, Vidgen HA, et al. CONSORT to community: translation of an RCT to a large-scale community intervention and learnings from evaluation of the upscaled program. BMC Public Health. 2017;17(1):918.

2. Leslie E, Magarey A, Olds T, Ratcliffe J, Jones M, Cobiac L. Community-based obesity prevention in Australia: background, methods and recruitment outcomes for the evaluation of the effectiveness of OPAL (Obesity Prevention and Lifestyle. Adv Pediatr Res. 2015;2(23):23.

3. Sutherland R, Campbell E, Nathan N, Wolfenden L, Lubans DR, Morgan PJ, et al. A cluster randomised trial of an intervention to increase the implementation of physical activity practices in secondary schools: study protocol for scaling up the Physical Activity 4 Everyone (PA4E1) program. BMC public health. 2019;19(1):883.

4. Stephanie Alexander Kitchen Garden Program t. N.D. [Available from: <https://www.kitchengardenfoundation.org.au/content/about-us>.

5. Hardy LL, King L, Kelly B, Farrell L, Howlett S. Munch and Move: evaluation of a preschool healthy eating and movement skill program. International Journal of Behavioral Nutrition and Physical Activity. 2010;7(1):80.

6. Morley B, Niven P, Dixon H, Swanson M, Szybiak M, Shilton T, et al. Population-based evaluation of the 'LiveLighter' healthy weight and lifestyle mass media campaign. Health education research. 2016;31(2):121-35.

7. Welsby D, Nguyen B, O'Hara BJ, Innes-Hughes C, Bauman A, Hardy LL. Process evaluation of an up-scaled community based child obesity treatment program: NSW Go4Fun(R). BMC Public Health. 2014;14:140.

8. Good Foundation T. Jamie Oliver's Learn Your Fruit and Veg N.D. [Available from: <https://www.jamiesministryoffood.com.au/jamie-olivers-learn-your-fruit-and-veg>.

9. Dunbar JA, Jayawardena A, Johnson G, Roger K, Timoshanko A, Versace VL, et al. Scaling Up Diabetes Prevention in Victoria, Australia: Policy Development, Implementation, and Evaluation. Diabetes Care. 2014;37(4):934-42.

10. Nathan N, Yoong SL, Sutherland R, Reilly K, Delaney T, Janssen L, et al. Effectiveness of a multicomponent intervention to enhance implementation of a healthy canteen policy in Australian primary schools: a randomised controlled trial. International Journal of behavioral nutrition and physical activity. 2016;13(1):106.

11. Eakin EG, Hayes SC, Haas MR, Reeves MM, Vardy JL, Boyle F, et al. Healthy Living after Cancer: a dissemination and implementation study evaluating a telephone-delivered healthy lifestyle program for cancer survivors. BMC cancer. 2015;15(1):992.

12. Duncan MJ, Brown WJ, Mummery WK, Vandelanotte C. 10,000 Steps Australia: a community-wide eHealth physical activity promotion programme. BMJ Publishing Group Ltd and British Association of Sport and Exercise Medicine; 2018.

13. Sahlqvist S, Veitch J, Abbott G, Salmon J, Garrard J, Acker F, et al. Impact of an Australian state-wide active travel campaign targeting primary schools. Preventive medicine reports. 2019;14:100866.

14. Merom D, Rissel C, Mahmic A, Bauman A. Process evaluation of the New South Wales Walk Safely to School Day. Health Promotion Journal of Australia. 2005;16(2):100-6.

15. Wutzke S, Morrice E, Benton M, Milat A, Russell L, Wilson A. Australia's National Partnership Agreement on Preventive Health: critical reflections from states and territories. Health Promotion Journal of Australia. 2018;29(3):228-35.

16. Horton P, Knijnik J, Clarke B. A Deleuzo-Guattarian ‘schizoanalysis’ of the Smart Moves–Physical Activity Program in Queensland State Schools. Journal of Human Sport and Exercise. 2014;9(3):668-85.

17. Ball K, Abbott G, Wilson MG, Chisholm M, Sahlqvist S. How to get a nation walking: reach, retention, participant characteristics and program implications of Heart Foundation Walking, a nationwide Australian community-based walking program. International Journal of Behavioral Nutrition and Physical Activity. 2017;14(1):161.

18. VicHealth. Be Active program evaluation highlights Victoria: VicHealth; 2015 [Available from: <https://www.vichealth.vic.gov.au/-/media/Images/VicHealth/Images-and-Files/Projects/Physical-Activity/Be-Active/VicHealth_Be_Active_evaluation_highlights.pdf?la=en&hash=8DFC7BA8CE72A159156E1C2FE64A224375280892>.

19. National Heart Foundation of Australia T. 25 years of Heart Foundation Tick 2014 [Available from: <https://www.heartfoundation.org.au/news/25-years-of-heart-foundation-tick>.

20. Penny B, Tuccia J, Brown MA. Beat it: Diabetes lifestyle and physical activity program-The effects and affordability of a 12-week community based, physical activity program for people with or at risk of diabetes. 2010.

21. Lonsdale C, Lubans D, Sanders T, Noetel M, McKay H, Morgan P, et al. Scaling-up an efficacious comprehensive school-based physical activity intervention: development, evaluation and dissemination of the iPLAY program. Journal of Science and Medicine in Sport. 2019;22:S14.

22. Nelson R. Active for life: A new VicHealth resource to shift thinking and get children more active. Australasian Parks and Leisure. 2014;17(3):20.

23. Hunter New England Local Health District T. Swap It 2018 [Available from: <http://www.goodforkids.nsw.gov.au/primary-schools/swap-it/>.

24. Healthy Kids Association T. Crunch & Sip N.D. [Available from: <http://healthy-kids.com.au/teachers/crunch-sip/>.

25. Department of Health T. Heartmoves 2012 [Available from: <https://www1.health.gov.au/internet/publications/publishing.nsf/Content/healthy-comm-lgag-att_c-toc~healthy-comm-lgag-att_c-heartmoves>.

26. Morgan PJ, Collins CE, Plotnikoff RC, Callister R, Burrows T, Fletcher R, et al. The ‘Healthy Dads, Healthy Kids’ community randomized controlled trial: A community-based healthy lifestyle program for fathers and their children. Preventive medicine. 2014;61:90-9.

27. SecondBite. SecondBite N.D. [Available from: <https://www.secondbite.org/>.

28. Department of Health & Human Services T. Healthy Together Victoria 2015 [Available from: <https://www2.health.vic.gov.au/about/publications/policiesandguidelines/What-is-Healthy-Together-Victoria>.

29. Good Foundation T. Jamie's Ministry of Food N.D. [Available from: <https://www.jamiesministryoffood.com.au/>.

30. Australian Government Department of Health T. Girls Make Your Move 2017 [Available from: <https://campaigns.health.gov.au/girlsmove/about-this-campaign>.

31. Department of Health T. 2010 National Healthy School Canteens Guidelines 2013 [Available from: <https://www1.health.gov.au/internet/main/publishing.nsf/Content/phd-nutrition-canteens>.

32. Health Star Rating system T. Health Star Rating system N.D. [Available from: <http://healthstarrating.gov.au/internet/healthstarrating/publishing.nsf/Content/Home>.

33. Department of Health T. Healthy Food Partnership N.D. [Available from: <https://www1.health.gov.au/internet/main/publishing.nsf/Content/Healthy-Food-Partnership-Home>.

34. Bravo A, Innes-Hughes C, O'Hara B, McGill B, Rissel C. Live Life Well@ School: evidence and evaluation summary 2008–2015. North Sydney: NSW Ministry of Health. 2016.

35. New South Wales Ministry of Health T. Finish with the right stuff N.D. [Available from: <https://www.rightstuff.health.nsw.gov.au/>.

36. Get Healthy Information & Coaching Service T. Get Healthy Information & Coaching Service 2020 [Available from: <https://www.gethealthynsw.com.au/health-professionals/about-the-service/>.

37. QCWA Country Kitchens T. Queensland Country Womens Aassociation Country Kitchens 2017 [Available from: <http://www.qcwa.org.au/countrykitchens/meet-the-team/what-we-do/>.

38. Foodbank Western Australia T. The Food Sensations® for Adults Program 2020 [Available from: <https://www.foodbank.org.au/WA/food-sensations-for-adults/?state=wa>.

39. Hetherington SA, Borodzicz JA, Shing CM. Assessing the real world effectiveness of the Healthy Eating Activity and Lifestyle (HEAL™) program. Health Promotion Journal of Australia. 2015;26(2):93-8.

40. Pettigrew S, Moore S, Pratt IS, Jongenelis M. Evaluation outcomes of a long-running adult nutrition education programme. Public health nutrition. 2016;19(4):743-52.

41. Yoong SL, Nathan N, Wolfenden L, Wiggers J, Reilly K, Oldmeadow C, et al. CAFÉ: a multicomponent audit and feedback intervention to improve implementation of healthy food policy in primary school canteens: a randomised controlled trial. international journal of behavioral nutrition and physical activity. 2016;13(1):126.

42. de Silva-Sanigorski A, Prosser L, Carpenter L, Honisett S, Gibbs L, Moodie M, et al. Evaluation of the childhood obesity prevention program Kids-'Go for your life'. BMC Public Health. 2010;10(1):288.

43. Laws R, Hesketh K, Ball K, Cooper C, Vrljic K, Campbell K. Translating an early childhood obesity prevention program for local community implementation: a case study of the Melbourne InFANT Program. BMC Public Health. 2015;16(1):1-15.

44. Hull NS, Schubert LC, Smith JP. Perspectives of key stakeholders and experts in infant feeding on the implementation of the Australian National Breastfeeding Strategy 2010-2015. Breastfeeding review : professional publication of the Nursing Mothers' Association of Australia. 2017;25(1):25-34.

45. Wiggers J, McElwaine K, Freund M, Campbell L, Bowman J, Wye P, et al. Increasing the provision of preventive care by community healthcare services: a stepped wedge implementation trial. Implementation Science. 2017;12(1):105.

46. Grunseit AC, Rowbotham S, Pescud M, Indig D, Wutzke S. Beyond fun runs and fruit bowls: an evaluation of the meso-level processes that shaped the Australian Healthy Workers Initiative. Health promotion journal of Australia : official journal of Australian Association of Health Promotion Professionals. 2016;27(3):251-8.

47. Farrell L, Lloyd B, Matthews R, Bravo A, Wiggers J, Rissel C. Applying a performance monitoring framework to increase reach and adoption of children's healthy eating and physical activity programs. Public Health Research & Practice. 2014;25(1):e2511408-e.

48. Dick M, Lee A, Bright M, Turner K, Edwards R, Dawson J, et al. Evaluation of implementation of a healthy food and drink supply strategy throughout the whole school environment in Queensland state schools, Australia. European journal of clinical nutrition. 2012;66(10):1124-9.

49. Shill J, Mavoa H, Allender S, Lawrence M, Sacks G, Peeters A, et al. Government regulation to promote healthy food environments--a view from inside state governments. Obesity reviews : an official journal of the International Association for the Study of Obesity. 2012;13(2):162-73.

50. Pratt S, DeMamiel M, Reye K, Huey A, Pope A. Food security in remote Indigenous communities. ANAO report no2; 2014.

51. YMCA Brisbane t. Schools' Breakfast Program 2018 [Available from: <https://www.ymcabrisbane.org/our-social-impact/schools-breakfast-program>.

52. Innes-Hughes C, Rissel C, Thomas M, Wolfenden L. Reflections on the NSW Healthy Children Initiative: a comprehensive state-delivered childhood obesity prevention initiative. Public Health Res Pract. 2019;29(1):e2911908.

53. Dunstan D, Cormick G, Wolf E. The Lift for Life community-based strength training program for people with or at risk of developing type 2 diabetes—A snapshot evaluation. Journal of Science and Medicine in Sport. 2010;12:e212.
