# Supplementary material for: Mechanisms of scaling up: combining a realist perspective and systems analysis to understand successfully scaled interventions
Source: Int J Behav Nutr Phys Act. 2021 Mar 22;18:42. doi: 10.1186/s12966-021-01103-0 (PMC7986035; doi:10.1186/s12966-021-01103-0)
Supplement: Supplementary file 3 — Additional file 3. Application of the WHO framework. Table of WHO ExpandNet framework principles and core areas, and their application to the qualitative (interview) and quantitative (survey) data collected. [file 12966_2021_1103_MOESM3_ESM.docx]

**Additional File 3. Application of the WHO ExpandNet framework for scaling up to data collection measures**

| **WHO ExpandNet principles & core areas** | **WHO ExpandNet principles & core areas sub-content** | **Data source** | |
| --- | --- | --- | --- |
|  |  | Survey | Interview |
| ***Principles*** |  |  |  |
| **1. Innovation design** | Components or practices are new/perceived as new in a particular context |  | X |
|  | Involves a set of interventions, including processes, necessary to build sustainable implementation capacities | - | - |
| **2. Successfully tested** | Intervention backed by locally generated evidence of program effectiveness | X | X |
|  | Feasibility obtained through pilot demonstration/experimental projects |  | X |
| **3. Involves deliberate efforts** | Involves a guided process (as opposed to spontaneous diffusion) | - | - |
| **4. Involves policy and program development on a lasting basis** | Importance of institutional capacity building and sustainability in scaling up | X |  |
|  | Developing, establishing political support | X |  |
|  | Developing, establishing managerial infrastructures | X |  |
|  | Developing, establishing human and budgetary resources and service components | X |  |
|  | Sustaining political support | X |  |
|  | Sustaining managerial infrastructures | X |  |
|  | Sustaining human and budgetary resources and service components | X |  |
| ***Core areas*** |  |  |  |
| **(1) Intervention attributes**  *Recommendations to maximise attributes of successful interventions* | Credible (e.g., based on sound evidence, advocated for by respected persons or institutions) | X | X |
|  | Observable (e.g., users can see results in practice) | - | - |
|  | Relevant (e.g., for addressing a persistent or sharply felt problem) | X | X |
|  | Relative advantage (e.g., greater advantage over existing practices, costs of implementation counteracted by the benefits) | X | X |
|  | Easy to install and understand (e.g., rather than complex and complicated) | X | X |
|  | Compatible (e.g., with potential users established values, norms, users’ facilities, fits well into practices of the national program) | X | X |
|  | Testable (e.g., without users having to completely adopt) | - | - |
|  | *Involve user org in a participatory process* |  | X |
|  | *Tailor the innovation to the context* |  | X |
|  | *Test the innovation under real-life operating conditions* | - | - |
|  | *Design research to test the innovation in light of the objectives of the project and decision-makers' expectations* |  | X |
|  | *Identify the key features central to success so the innovation can be streamlined and more readily replicated during scale up* |  | X |
|  | *Reflect on the degree and nature of change that the innovation implies for the user organisation* | - | - |
|  | *Initiate scaling up after the effectiveness and feasibility of the innovation have been established* |  | X |
| **(2) User organisation(s) attributes**  *Recommendations to enhance capacity of user organisation(s) to enhance scale up* | Members of user organisation(s) perceive a need for innovation | X |  |
|  | User org has appropriate implementation capacity | X | X |
|  | Timing and circumstances are right | X |  |
|  | User org possesses effective leadership and internal advocacy | X |  |
|  | The resources and user organisations are compatible | - | - |
|  | *Recognise the value of policy entrepreneurs and champions* |  | X |
|  | *Assess strengths and weaknesses of the user organisation(s) and develop strategies to build capacity* |  | X |
|  | *Make use of existing processes and structures* |  | X |
|  | *Acknowledge scaling up may be an institutional change task of major proportions* |  | X |
| **(3) Environment attributes**  *Recommended ways of maximising opportunities for sustainable scale up* | *Identify environmental factors influencing scale up and understand how they affect the process* |  | X |
|  | *Make timely use of opportunities arising in environment to enhance positive supports for scale up* | - | - |
|  | *Continue to assess changes in environment as the process of scaling up evolves* |  | X |
| **(4) Resource team attributes**  *Recommendations to ensure resource team can maximise potential sustainable scale up* | Effective and motivated leaders who command authority and have credibility with user organisation |  | X |
|  | A unifying vision | - | - |
|  | Understand the political, social and cultural environment in which scaling up takes place | X |  |
|  | Ability to generate financial and technical resources | X |  |
|  | In-depth understanding of the user organisation capacities and limitations | X |  |
|  | Relevant technical skills, including research and evaluation skills | X |  |
|  | Capacity to train members of user organisation | X |  |
|  | Capacity to assist the user org with management intervention needed to implement the innovation | - | - |
|  | Skills and experience with scaling up | X |  |
|  | Compatibility with user organisation | - | - |
|  | *Include individuals who have been part of the design and testing of the intervention* | X |  |
|  | *Involve members of user organisation(s)* | X |  |
|  | *Locate the resource team as closely to user organisation as possible* |  | X |
|  | *Ensure the team have the necessary skills and capabilities* | X |  |
|  | *Anticipate the need to augment and adapt the resource team as up proceeds* | - | - |
|  | *Support user organisation ownership of the intervention and process* | X |  |
| **(5) Scale up strategy** | Addresses both horizontal expansion of the innovation and vertical scaling up to ensure sustainability | X | X |
|  | Ensure scaling up is proceeding smoothly before adding new innovations | - | - |
|  | Use multiple channels to tell a compelling story | X | X |
|  | Build coalitions and networks (for program and policy advocacy) | X |  |
|  | Organise training strategies to address both content and process in scaling up | - | - |
|  | Make the most of demonstration sites (e.g., pilot sites) |  | X |
|  | Create opportunities for ongoing learning | X |  |
|  | Organising the scaling up process |  | X |
|  | Assessing the cost of and mobilising of resources |  | X |
|  | Monitoring and evaluation | X | X |
| **(6) Planning and management**  *Recommendations for the strategic planning and management of the scaling up process* | *Watch for correct and incorrect imbalances as elements of the scaling up system interact* | - | - |
|  | *Recognise trade-offs are necessary* | - | - |
|  | *Commit to upholding participation of a broad range of stakeholders* | - | - |
|  | *Protect intervention elements that differ most from user organisation culture (e.g., those most easily lost during scale up)* | - | - |
|  | *Maintain the resource team staying power* | - | - |
|  | *Remain vigilant (e.g., expect unexpected and be prepared to act quickly or pause momentarily)* | - | - |

No data source indicated means that the core area not directly asked via survey/interview question
