# Supplementary material for: Mechanisms of scaling up: combining a realist perspective and systems analysis to understand successfully scaled interventions
Source: Int J Behav Nutr Phys Act. 2021 Mar 22;18:42. doi: 10.1186/s12966-021-01103-0 (PMC7986035; doi:10.1186/s12966-021-01103-0)
Supplement: Supplementary file 4 — Additional file 4. Mechanisms underpinning scale up. Table listing the 53 commonly occurring mechanisms underpinning scale up, and their relationship to the eight key outcomes. [file 12966_2021_1103_MOESM4_ESM.docx]

**Additional File 4. Summary of commonly occurring mechanisms linked to outcomes for successful scale up**

| **Outcomes for successful scale up** | **^b^Mechanism variable label** | **Mechanism description**  **(^a^n=53)** |
| --- | --- | --- |
| 1. **Community adoption** | Community buy-in/perceived value | 1. Community see value and embed intervention in practice |
|  | Perceived relative advantage over existing practices | 1. Community see advantage over existing practices |
|  | Strategic targeting dissemination channels/networks | 1. Public promotion and dissemination of intervention through strategic stakeholder channels leading to increased legitimacy |
|  | Likelihood of addressing stakeholder goals/ priorities | 1. Stakeholders see advantage over existing practices/way to meet their agenda/ targets/goals |
|  | Perceived credibility/legitimacy to solve problem | 1. Increased intervention visibility in community among government to enhance perceived legitimacy and impact of advocacy efforts |
|  | Perceived relative advantage over existing practices | 1. Demonstrable evidence of intervention performance in real-world increases credibility and legitimacy |
|  | Gov./advocates awareness/value & support | 1. Politically well-connected advocates inspiring others to support scale up and timing |
| 1. **Political support/ buy-in** | Aligned with state/national priorities | 1. Intervention objectives align with or produces evidence used for national strategies/ policies |
|  | Gov./advocates awareness/value & support | 1. Politically well-connected advocates inspiring others to support scale up and timing |
|  | ^a^Involvement of political advocates & strategies for political support | 1. Leveraging opportune moments and advocacy strategies build political support |
|  | Perceived credibility legitimacy to solve problem | 1. Favourable politically despite lack of evidence for impact on target outcome; garners ongoing support/funding |
|  | ^a^Impact of political instability | 1. Political instability undermines/ends political advocacy and resource availability in government; intervention no longer valued/prioritised |
|  | Early engagement of political advocates in gov. (health/non-health) | 1. Advocates identified early; at various levels of government, within and outside of health system |
|  | Inclusion of resources for stakeholder engagement | 1. Resources (e.g. time and funds) required to engage with government planned for and strategies embedded in scale up approach |
|  | Inclusion of resources for stakeholder engagement | 1. Adoption of a bi-partisan approach to advocacy in lieu of government/political changes |
|  | Diversity of support/ resources (gov./non-gov.) | 1. Diversification of funds to ensure ongoing stakeholder commitment to implementation |
|  | Gov./advocates awareness/value & support | 1. High awareness and value placed on intervention among key political actors/ advocates |
|  | Inclusion of resources for stakeholder engagement | 1. Poor understanding of government/political structures; resources (time and funds) required for scaling with governments unanticipated and excluded from scale up approach |
|  | Perceived credibility/legitimacy to solve problem | 1. Intervention *initially* politically favourable (valued and prioritised) at all government levels; need not be sustained support |
| 1. **Institutional implementation capacity** | Implementation of 'parallel'/conflicting processes | 1. Strategies to address implementation weaknesses *within* scale up planning; to avoid 'parallel' processes |
|  | Ability to leverage existing processes/structures | 1. Strategies to overcome barriers and leverage facilitators to effective implementation introduced on an *ad hoc basis* |
|  | Planning/delivery of capacity building as required | 1. Capacity building strategies or incentives introduced retrospectively overtime to overcome/minimise impact of implementation weaknesses |
|  | Planning & delivery of capacity building as required | 1. Resources or incentives introduced *during* scale up to overcome weaknesses or minimise their impact, enhancing implementation. |
| 1. **System-level (policy) embeddedness** | System integration, contextual relevance & local resource mobilisation | 1. Combined integration into structure, budgets and practices of system (centralised approach) whilst contextually relevant and involving local mobilisation of resources (decentralised approach) |
|  | Conflict/ownership changes between gov. levels | 1. Conflict between centralised and decentralised approaches impacts mobilisation of resources and 'ownership'; reduces integration into systems, budgets and practices |
| 1. **Replication in other contexts** | ^a^Intervention/implementation applicable range of settings | 1. Intervention and implementation resources easily replicable and/or facilitate flexibility in different settings |
|  | Understanding of key intervention/implementation components | 1. Staggered implementation and scale up of critical components, to establish sustainability and lasting institutional capacities at all levels |
|  | ^a^Key components & barriers/facilitators known | 1. Key features central to success (i.e. context of testing, underlying intervention concepts) understood before roll out; not necessarily previously tested in the scale up context |
|  | Availability resources/strategies for contextual adaptation | 1. Resources/strategies put in place to enable contextual adaptation |
|  | Fidelity of implementation components | 1. Components essential for effectiveness and fidelity of implementation during replication remain intact during scale up |
|  | Planning for implementation barriers across settings | 1. Impact implementation barriers across different settings planned for/minimised |
| 1. **Stakeholder buy-in/ perceived value** | Perceived priority/ problem on stakeholder agenda | 1. Strong perceived need/on the agenda of stakeholders to meet a priority area. Stakeholders see advantage over existing practices/way to meet their agenda/ targets/goals |
|  | Perceived credibility/legitimacy to solve problem | 1. Intervention perceived as credible/legitimate and more likely to solve the problem |
|  | Perceived credibility/legitimacy to solve problem | 1. Lack of better/alternative approach; intervention perceived as credible/legitimate despite a lack of evidence base |
|  | Fidelity of implementation components | 1. Components essential for effectiveness and fidelity of implementation during replication remain intact during scale up |
|  | Perceived data/methodology credibility | 1. Data collection (prospective and retrospective) tailored to evidence perceived as credible by stakeholders |
|  | Understanding of key intervention/ implementation components | 1. Staggered implementation and scale up of critical components, to establish sustainability and lasting institutional capacities at all levels |
|  | Perceived relative advantage over existing practices | 1. Demonstrable evidence of intervention performance in real-world increases credibility and legitimacy |
|  | Spontaneous scaling | 1. Spontaneous scaling in response to government targets/pressure for roll-out; inadequate resources or planning |
|  | Targeted messaging to different stakeholders & audiences | 1. Intervention messaging/goals framed according to values and needs of different audiences, reaching beyond health sector and hard to reach groups |
|  | Targeted messaging to different stakeholders & audiences | 1. Data collected modified over time to reflect changes in Government needs |
|  | ^a^Key components & barriers/facilitators known | 1. Key features central to success (i.e. context of testing, underlying intervention concepts) understood before roll out; not necessarily previously tested in the scale up context |
| 1. **Evidence for impact on target outcome** | Evidence perceived important by stakeholders considered | 1. Stakeholder perceptions of critical/persuasive evidence included in evaluation plan, independent of researcher recommendations |
|  | Stakeholder’s value formal evaluation/data | 1. Ongoing evaluation less relevant at point of scale up; prior (early) evaluations used to justify political decision making |
|  | Spontaneous scaling | 1. Spontaneous scaling in response to government targets/pressure for roll-out; inadequate resources or planning |
| 1. **Community sustainability/ embeddedness** | Early identification of successful intervention/implementation attributes | 1. Attributes of future success and potential failure may/may not be identified early so an ‘improved’ intervention is scaled up |
|  | Likelihood of addressing stakeholder goals/priorities | 1. Intervention and implementation process aligns with stakeholder priorities/objectives and community context/values |
|  | Prior development intervention/scale up resources | 1. Scale up resources (physical and fiscal) required from community/Government explicit and planned for |
|  | Prior development intervention scale up resources | 1. Required scale up resources (physical and fiscal) developed as scale up unfolds; reactive to real-world implementation |
|  | Availability resources/strategies for contextual adaptation | 1. Resources/strategies put in place to enable contextual adaptation |
|  | Planning for implementation barriers across settings | 1. Impact implementation barriers across different settings planned for/minimised |
|  | Relevance to multiple goals/priorities of target systems | 1. Intervention meets multiple system goals/priorities and valued/relevant to multi-sector stakeholder agendas |
|  | ^a^Involvement of political advocates & strategies for political support | 1. Leveraging opportune moments and advocacy strategies build political support |
|  | Perceived credibility/legitimacy to solve problem | 1. Favourable politically despite lack of evidence for impact on target outcome; garners ongoing support/funding |
|  | ^a^Key components & barriers/facilitators known | 1. Intervention more applicable to a wide range of settings/contexts (i.e. compatible with varying established norms) |
|  | Capitalisation of existing setting resources | 1. Intervention capitalises on existing delivery system structures, increase integration into existing policies/practices |
|  | ^a^Involvement of political advocates & strategies for political support | 1. Resources generated and/or time taken to engage with respected/influential community members and organisations |
|  | Responsiveness to community need & political system changes | 1. Intervention and scale up responsive to real-world conditions, reflecting community need and changes in political environment |
|  | Prior awareness/planning for scaling costs | 1. Costs of scaling (community/government funds required) need not be known (explicit) or planned for in advance; influenced by political favourability |
|  | Scale up responsiveness to implementation | 1. Evaluation data used to adjust to the scale up process or resources needed over time; increasing responsiveness to real-world implementation and potential practice/policy changes |
|  | Formal measures and evidence of impact | 1. Evaluation not embedded in scale up plan, monitoring/visual accounts replace formal measure of impact; leads to retrospective evaluation to meet stakeholder's needs |
|  | Stakeholder’s value formal evaluation/data | 1. Evaluation not embedded in scale up plan; increased importance/perceived power of visual evidence ('individual experiences') |
|  | Stakeholder’s value formal evaluation/data | 1. Evaluation undertaken as planned; stakeholder perceptions of data value/relevance/influence/persuasiveness affected use of the data to inform practice/policy |
|  | Political influence/funding cuts | 1. Evaluation embedded in scale up, changes/ceases due to political instability/funding cuts; data unable to influence practice/policy |
|  | Political influence/funding cuts | 1. Evaluation embedded in scale up, and monitoring/data collection processes primarily driven/influenced by government/stakeholders as opposed to researchers; can hinder transparency and influence on practice/policy |

^a^Mechanism also features as a ‘Context’ for scaling up as shown in Fig. 4 Complex Systems Model of Scaling Up. ^b^Mechanism variable label as shown in Fig. 4 Complex Systems Model of Scaling Up
